# Influence of Cognitive Orientation and Attentional Focus on Pain Perception

**DOI:** 10.3390/ijerph18137176

**Published:** 2021-07-05

**Authors:** Pierluigi Diotaiuti, Stefano Corrado, Stefania Mancone, Lavinia Falese, Angelo Rodio, Thaìs Cristina Siqueira, Alexandro Andrade

**Affiliations:** 1 Department of Human Sciences, Society and Health, University of Cassino and Southern Lazio, 03043 Cassino, Italy; stefano.corrado@unicas.it (S.C.); s.mancone@unicas.it (S.M.); l.falese@unicas.it (L.F.); a.rodio@unicas.it (A.R.); 2 Health and Sports Science Center, Department of Physical Education, CEFID, Santa Catarina State University, Florianópolis 88035-901, Brazil; thais.siqueira@udesc.br (T.C.S.); alexandro.andrade.phd@gmail.com (A.A.)

**Keywords:** catastrophizing, psychophysiology, psychometrics/testing, temperature, pain tolerance, cold pressor test, methods of pain evaluation

## Abstract

Background. Recently, a growing interest has emerged in the role of attention and hypervigilance in the experience of pain. Shifting attention away from pain seems likely to reduce the perception of pain itself. Objectives. The present study has been designed to test the following overall hypotheses: (1) disposition to catastrophize, self-efficacy perceived in pain resistance (task self-efficacy), previous experiences concerning the tolerance of physical pain, and degree of impulsiveness are significant predictors of the decision to abandon a painful test such as the cold pressor test (CPT); (2) the manipulation of the attentive focus (internal or external) can influence the level of perceived pain. Methods. Effects of the manipulation of attentional focus (internal and external) on pain perception and response of trial abandonment were evaluated in a sample of university students (*n* = 246) subjected to the cold pressor test. Results. A significant effect (*p* < 0.05) was found through a test–retest comparison on the final level of perceived pain among subjects who had received instruction to externalize the focus of their attention (mixed factorial analysis of variance), but no significance was observed with respect to the decision to abandon the experiment. A general explanatory model of the abandonment behavior demonstrating overall good fit measurements was tested too. Conclusion. The abandonment of tests has been shown to be predicted mainly by catastrophic attitude. Attentive impulsiveness showed a further positive effect on catastrophic attitude. Perceived self-efficacy in the tolerance of pain limited learned helplessness, which in turn positively influenced catastrophizing.

## 1. Introduction

The role of attention and hypervigilance in the experience of pain has been addressed in several recent publications [1,2]. Since pain is physiologically an alarm signal, the demand for attention is due to the need to modify functional patterns in order to escape or avoid tissue damage [3]. When pain, especially pain with neuropathic origins, becomes chronic, usually after 3 months, it can bring poor quality of life, anxiety, depression and sleep disorders, and regular treatment useful for nociceptive pain such as analgesics does not usually work [4], with a consequent constant focus on the pain. Therefore, it is in our best interest to be able to turn our attention elsewhere, implementing the mechanism of distraction, and carry out our usual activities [5,6]. Shifting attention away from pain generally reduces the perception of pain itself; this modulatory effect involves the descending inhibitory nerve pathways that terminate at the level of the posterior spinal cord [7]. However, people with chronic pain seem to show hypervigilance towards pain; this distortion of attention is involuntary and resists distraction strategies and contributes to the crystallization of the condition of pain and suffering, in a vicious circle of functional limitation, fear, and depression [8,9].

From a neuropsychological point of view, attention can be considered as a function that regulates human cognitive activity, which is the processing of information from the internal and external environment through the selection, filtering, and organization of information material in order to provide adequate responses to the environment. In the attentional process, several components come into play: excitement, selective attention, hypervigilance, or sustained attention [6].

There are numerous studies on the effects of attention and distraction on the experience of pain and they report variable results: some report excellent pain relief [10,11], while others deny this result [12].

According to Dragustin, Pavin and Kotzumuth [13], the possible explanations imply that the relationship between attention and the perception of pain is a complex phenomenon and that an approach to evaluate their interaction is very important in investigating the role of cognitive factors in pain modulation. It is very likely that no psychological factor alone contributes to the possible analgesic effect on pain perception, but only certain combinations of these factors can have that effect. In addition, the variability of results in the different studies can be partly attributed to the lack of or insufficient control of the state of attention of the subjects involved in the various experiences [14].

Individuals’ beliefs and expectations can influence the perception of pain and the responses to pain [15]. The individuals’ responses to pain can also be influenced by pain catastrophizing, the process during which pain is interpreted as being extremely threatening [16]. Possible responses include fear-avoidance or endurance models based on different cognitive mechanisms and patterns [15]. Hasenbring and Verbund [15] proposed that fear-avoidance responses consist of fear/anxiety associated with automatic thoughts (e.g., catastrophizing) or more generalized appraisals (e.g., fear-avoidance beliefs) on the cognitive level, and the avoidance of pain-associated activities on the behavioral level. In contrast, endurance responses refer to thoughts of suppression, distraction from pain, or minimization, with task persistence behavior in spite of severe pain [17].

Chronic and acute pain have a different nature and require different assessment and management approaches [18,19,20]. Acute pain can be assessed through several reliable and valid self-report scales such as the visual analogue scale (VAS) and the verbal rating scale (VRS-4) reporting “current” pain intensity or pain intensity “in the last 24 h” [20], or using specific physical and mechanical stimuli sensory perception methods such as the pressure pain threshold (PPT), useful for large cohort studies but prone to several possible biases [21,22], and the cold pressor test (CPT) [23,24]. The CPT consists of the immersion of one hand in ice water for a specific amount of time according to the perception of pain and response of the autonomous nervous system. The CPT has been widely used to evaluate experimentally induced pain and chronic pain [24]. The test allows the measurement of the differences between individuals and, when used in combination with other tools such as specific psychological scales and/or clinical status, it can give information on the model undergoing the process of pain.

To our knowledge, no studies have previously been conducted to understand the factors that may predict the abandonment of experimental tests with painful content for the subject. The theoretical model of reference for this study is precisely the avoidance–endurance response model of perceived pain. The present study aimed to assess to what extent the abandonment (giving up) versus endurance behavior could be associated with specific psychological variables (impulsivity, catastrophizing, self-efficacy), and further, to verify whether the manipulation of the attentional focus (external/internal) could influence the intensity of perceived pain, and, therefore, affect the abandonment/resistance.

The present study has been designed to test the following overall hypotheses: (1) disposition to catastrophize, the self-efficacy perceived in pain resistance (task self-efficacy), previous experiences concerning the tolerance of physical pain, and the degree of impulsiveness are significant predictors of the decision to abandon a painful test such as the cold pressor test (CPT); (2) the manipulation of the attentive focus (internal or external) can influence the level of perceived pain; (3) the adequacy of a path analysis (structural equation modeling) in illustrating the relationships and influences between the variables identified in predicting the experimental abandonment behavior.

## 2. Materials and Methods

### 2.1. Study Design

This study was characterized as a randomized clinical trial. The randomized controlled trials registration number is 2021-003638-35.

### 2.2. Participants

**Figure 1:**
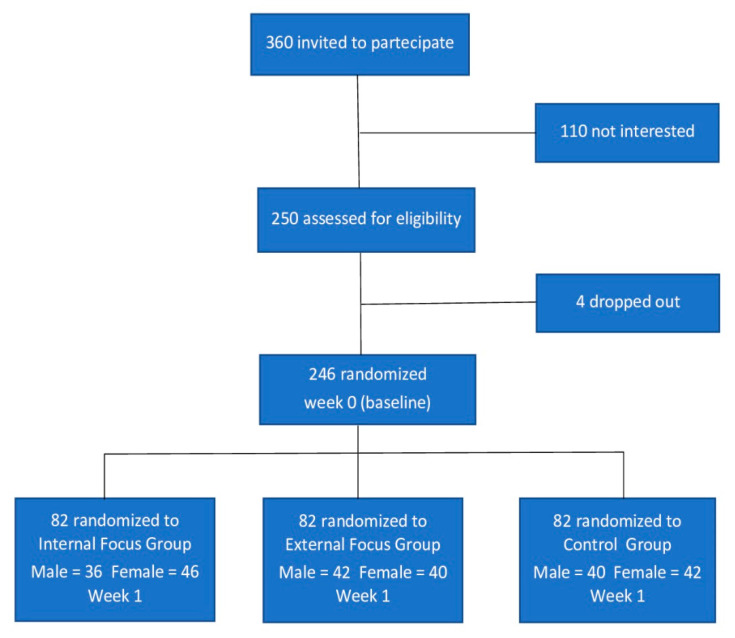
Consort diagram of trial enrollment and flow of the study.

The sampling was intentionally non-probabilistic. The study involved 246 university students recruited in the trials: 118 (48%) males, 128 (52%) females and M_age_ = 23.45 DS = 3.27. The sample recruitment procedure was through an open invitation to students attending the Psychology of Personality and Sport Psychology courses. Inclusion criteria were: (1) age range of 18–28 years, (2) no history of musculoskeletal pain requiring healthcare within the preceding 3 months, (3) no musculoskeletal pain at the time of testing and (4) ability to lie in a prone position for at least 30 min without discomfort. Exclusion criteria were: (1) inability to understand and follow instructions in verbal and written Italian, (2) any health condition potentially causing sensory deficits, such as diabetes mellitus or neurological disorders, (3) any history of chemotherapy, (4) currently taking medication that can affect sensation, and (5) currently pregnant. Participants were asked to limit intake of caffeine, alcohol and any medication that can cause sleepiness or analgesia for the 24-h prior to each testing session. The procedure was explained and written informed consent was obtained before data collection commenced. Of 360 total students, 250 indicated their willingness to participate in the study; of these, 4 subsequently dropped out before the start of the trial. Therefore, 246 was the final number of participants: male = 118 (48%); female = 128 (52%); M_age_ = 23.64 SD = 43.87. In order to control the covariate of sex, a stratified randomized distribution in three groups (two experimental and one control) was subsequently performed. Testing the final distribution between groups confirmed the absence of significant differences (*p* > 0.05). The following Consort diagram (Figure 1) shows the whole trial enrollment and flow of the study.

### 2.3. Procedures

The study was approved by the Institutional Review Board of the University of Cassino and Southern Lazio (IRB_SUSS_06:13/02/19). The participants (all volunteers) were each summoned to the laboratory twice, at an interval of a week. The first time, they: (a) received information on the study from the investigators, (b) provided informed consent to participate and were informed about the safety on the scientific and aggregate use of the data provided, in accordance with the Declaration of Helsinki, (c) completed a preliminary questionnaire for the collection of demographic and psychological data, and (d) carried out the first session of the cold pressor test (CPT). The CPT was adopted as a method to induce and measure variations in pain perception, recording any decision by the participants to abandon the experimental test.

Before the execution of the CPT, the protocol included an acclimatization phase in which the participants were asked to immerse their non-dominant hand inside a basin containing three liters of water at room temperature for two minutes, both in order to accustom the hand to low temperatures and to make the basal temperatures for the participants homogeneous before the test.

The participant was then asked to place his/her non-dominant hand and wrist in 8 °C water in a 13-L plexi-glass container connected with a circulating water bath (Julabo PF40-HE) and maintain it there as long as he/she could or until the maximum time of 90 s was reached. For safety reasons, the test was terminated after 1.5 min (90 s) if the participant had not already removed his/her hand. Although the maximum immersion limit of 3–5 min is normally applied to healthy adults, the test limit of 1.5 min was chosen to limit the risk of tissue injury [25,26,27,28].

The perception of the pain component was evaluated by scoring on a 10-interval numerical graduation scale (0 = no perceived pain, 10 = maximum perceived pain). In our case, as pain stimulus, the subject was required to immerse their non-dominant hand and indicate with the index of the other hand the progression of painful perception from the initial to the final moment of the test (90 s). However, all participants were given the option to withdraw the hand and interrupt the test when the discomfort was considered unbearable. The experimenter recorded the starting and ending time with a stopwatch, as well as the time of the subject’s voluntary withdrawal from the test. A portable camera placed on a support at the back of the subject allowed the recording of the values of the progression of perception indicated on the paper on a scale of 1–10. The stability of the temperature of water in the tank was also controlled with the help of an internal thermometer and using a KK Moon IR external infrared thermometer device. A maximum temperature fluctuation of 3 °C was allowed in the tank.

During the second visit, after a week (re-test), the experimental manipulation of attentional focus was achieved through specific instruction. Participants were randomized into three groups of 82 subjects each.

The first group was asked to perform the CPT again but to continuously focus attention on their immersed hand during the immersion (internal focus).

The second group was asked to perform the CPT again but to continuously focus attention on a warm outdoor scene (lying on the beach at noon in the summer sun) during the immersion (external focus).

The third group was not given specific instruction to focus their attention and repeated the same procedure performed in the first session (control group without focus instruction).

At the end of the trial, the first and second groups were asked to indicate on a scale of 1 to 5 points how well they had managed to stay concentrated on the focus task.

### 2.4. Instruments

For the CPT, a Termocriostat Julabo (CF40-HE, Julabo GmbH, Seelbach, Germany) was used; an external infrared thermometer (IR, KKmoon, Guangdong, China), LCD thermometer with suction cup (Ueetek, Shenzhen, China), and a video camera (Camcorder FHD 1080P, Panasonic, Kadoma, Japan) were also employed for recording the experiment. A general questionnaire covering a demographic and psychometric section was used to record the responses of participants. The latter included: (a) Barratt Impulsiveness Scale-11 (BIS-11) [29,30], a 30-item self-report questionnaire with Likert scale from 1 (rarely) to 4 (almost always/always) designed to evaluate general impulsiveness, taking into consideration the multifactorial nature of the construct. The participant was asked to indicate the frequency with which he/she reacts in a certain way to particular situations. The structure of the instrument allows the evaluation of six first-order factors (attention, motor, self-control, cognitive complexity, perseverance, cognitive instability) and three second-order factors (attentional impulsiveness, motor impulsiveness, non-planning impulsiveness). The total score ranges from 30 to 120 and higher scores indicate greater impulsivity. In the present study, the Italian validated version of this questionnaire was used and had a Cronbach’s alpha reliability score of 0.74 [31]. Furthermore, (b) Pain Catastrophizing Scale (PCS) was also used, which was developed to help quantifying an individual’s pain experience with questions about how they feel and what they think about when they are in pain [32,33]. This is a self-administered clinical scale of 13 items with a score assigned on a scale from 0 (never) to 4 (always) measuring three different variables: rumination, magnification (“amplification”), and sense of powerlessness (“helpless34ness”). Pain catastrophizing is characterized by the tendency to magnify the threat value of a pain stimulus and to feel helpless in the presence of pain, as well as by a relative inability to prevent or inhibit pain-related thoughts in anticipation of, during, or following a painful event. Higher scores indicate higher levels of pain-related anxiety. The following pain anxiety severity levels have been recommended for clinical interpretation: mild = 0 to 34; moderate = 35 to 67; and severe = 68 to 100. The tool showed a Cronbach’s alpha reliability score of 0.83. Finally, a four-item self-efficacy scale, the Task-Specific Self-Efficacy Scale (c), was created by the authors to examine expectations about coping with the CPT. The items, rated on a 7-point scale ranging from not at all to very much, assessed participants’ degree of certainty that they would be able to control the pain associated with the CPT, cope well during the CPT, perform the experimental task successfully, and not be able to manage the pain related to the CPT (reverse-scored). Alpha coefficient for these four items was 0.91 in this study.

### 2.5. Statistical Analysis

The *t*-test, chi-square test, mixed factorial analysis of variance, univariate analysis of variance, hierarchical regression, and structural equation modeling were used for statistical analyses of the data. To test the adequacy of the model, as also suggested by Teo [34], the following fit indices were considered: (1) the chi-square; (2) CFI (Comparative Fit Index); (3) TLI (Tucker Lewis Index); (4) RMSEA (Root-Mean-Square Error of Approximation), with CFI and TLI > 0.95 and RMSEA < 0.06 as excellent model fit indicators [35].

## 3. Results

### 3.1. Descriptive Analysis of Experimental Abandonments

With reference to the test sessions using the CPT (test and re-test), the dropouts recorded had the following distribution: 19.5% (*n* = 48) abandoned both trials, 8.9% (*n* = 22) abandoned only the first trial, 5.7% (*n* = 14) abandoned only the second trial. Of the latter, five belonged to the internal focus group, two to the external focus group, and seven to the control group. The remaining 65.9% (*n* = 162) completed both trials on schedule. On evaluation of the difference in abandonment with respect to gender, no significance emerged using the χ^2^ test (*p* > 0.05). For the purpose of the subsequent comparative evaluations, the following conditions described above were considered.

### 3.2. Trial Abandonment and Individual Differences

Psychological dimensions that were significantly associated with the abandonment of the first, second, and both CPTs are shown in Table 1.

A hierarchical regression was performed in order to evaluate if dispositional variables can be predictive of trial abandonment. The preliminary verifications of the regression assumptions excluded the presence of multivariate outliers. In the three cases of abandonment, the individual disposition to catastrophize was the only significant predictor. The respective outcomes are shown in Table 2.

### 3.3. Dispositional Factors and Pain Perception

The possibility of a significant association between dispositional factors (impulsiveness, catastrophizing, perceived self-efficacy in resistance) and levels of perceived pain at the beginning and end of the first trial (without manipulation of the attentional focus) was verified, evaluated by scoring on a 10-interval numerical graduation scale (0 = no perceived pain, 10 = maximum perceived pain). Only the measurement of motor impulsiveness showed, with the univariate analysis of variance test, a significantly higher average score of perceived pain at the end of the trial in the subjects with a higher level of motor impulsivity: F (1245) = 5.802, *p* = 0.03, *η^2^* = 0.06, OP = 0.790.

### 3.4. Effects of Focus Manipulation on Perception of Pain

As indicated in the procedural description, during re-test (second trial), the participants were randomly distributed into three groups, of which the first two acquired experimental status by virtue of the manipulation of the attentive focus, administered as a preliminary instruction to the test: request to internalize the focus on their own immersed hand for the first group; outsourcing request with the display of a scene from the opposite valence (hot) for the second group; no instructions for the third group, which served as a control. At the end of the trial, all members of the first and second groups were asked to indicate on a scale of 1 to 5 how well they had managed to stay concentrated on their focus task. The mean of the responses of the participants in the two groups confirmed that there were no particular difficulties to attend the focus task (M_1_ = 4.7 SD = 0.30; M_2_ = 4.5 SD = 0.23).

An analysis of variance with a mixed factorial model was established, which included the time variable (test–retest) as a factor within the participants and the group variable as a factor between the subjects. The group of those who abandoned the test (*n* = 62) was excluded from the analysis in order to be able to measure the manipulation effect of the focus on pain perception in the subjects who completed the task assigned during re-test (*n* = 184). Mauchly’s test of sphericity indicated that the assumption of sphericity was met for the two-way interaction, χ^2^(2) = 3.93, *p* = 0.075. There was no statistically significant interaction between group and time (test/retest) on perceived pain, F(4, 181) = 4.138, *p* = 0.124, partial η^2^ = 0.046. The main effect of time showed a statistically significant difference in mean pain perception at different time points, F(1, 181) = 146.75, *p* < 0.001, partial η^2^ = 0.622. The main effect of group showed that there was a statistically significant difference in mean pain perception between groups, F(2, 181) = 6.694, *p* = 0.002, partial η^2^ = 0.131.

**Figure 2:**
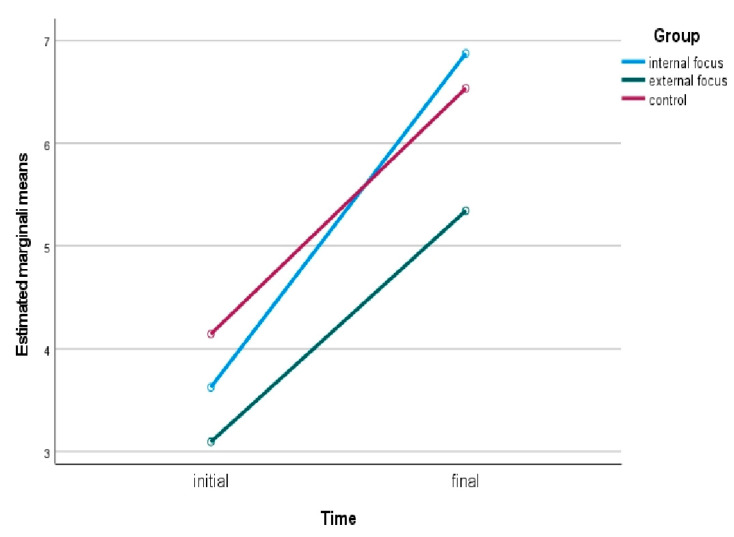
Initial and final pain perception among the groups.

The results showed that participants belonging to the group that received the instruction to externalize their focus of attention, had a significantly lower average level of perceived pain (*M* = 4.22, *SE* = 0.24, *CI 95%* 3.75; 4.69) compared to the group given the instruction to internalize their focus of attention (*M* = 5.25, *SE* = 0.24, *CI 95%* 4.78; 5.72) and to the control group (*M* = 5.34, *SE* = 0.25, *CI 95%* 4.84; 5.84). The overall change in pain perception in the three groups in relation to the two measurement moments (initial and final) of the trial is shown in Figure 2 below.

### 3.5. A Model of Path Analysis for Experimental Abandonment

**Figure 3:**
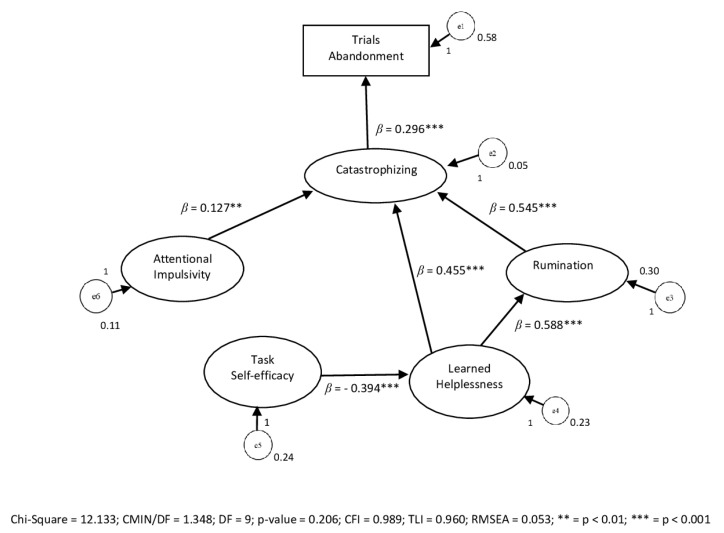
Path model.

A general structural model of path analysis was subsequently constructed, considering impulsiveness, task self-efficacy, disposition to catastrophizing, disposition to rumination, previous negative experiences (learned helplessness), and the frequency of abandonment. For the construction of this model, the abandonment data of the first trial were considered, thus excluding the variable of the attentional focus, which was present only in the second trial. The SPSS Amos software version 22 (IBM, USA) was used for data processing. The model showed overall good fit measurements: χ^2^ = 12.133; CFI = 0.989; TLI = 0.960; RMSEA = 0.053. As shown in Figure 3, at the proximal level, catastrophizing appeared as the direct predictor of the choice to abandon the trials (with a standardized estimate of the regression weight of 0.296 for *p* < 0.001). At the distal level, previous experiences (learned helplessness) act directly on the tendency to catastrophize (with standardized estimation of the regression weight 0.455 for *p* < 0.001) and indirectly stimulate the rumination tendency (with standardized estimate of the regression weight 0.588 for *p* < 0.001), which constantly reminds the subject of memories associated with unpleasant and suffering sensations. All this leads to amplifying and overestimating the dangerousness of the situation by anticipating negative outcomes, thus influencing the subject’s catastrophizing (with a standardized estimate of the regression weight 0.545 for *p* < 0.001). Further, once again at a distal level, the subjective estimate of self-efficacy in pain tolerance was shown to be a negative influence on learned powerlessness, i.e., the weight of memories associated with unpleasant and suffering sensations (with standardized estimation of regression weight −0.394 for *p* < 0.001). This effect was particularly relevant, considering that learned powerlessness has a decisive role in increasing both the ruminative and catastrophic component.

Moreover, with regard to catastrophizing, the influence of impulsiveness was also significant in the model (with a standardized estimate of the regression weight 0.127 for *p* < 0.01). The respective outcomes are shown in Table 3.

## 4. Discussion

The objective of our study was to understand which psychological aspects could lead the participants to abandon the experimental test, considering it unbearable. Beyond what could be considered a subjective sensitivity to the source of pain induced by trials (the cold), the disposition to catastrophize in its components of rumination, amplification, and learned helplessness, as assessed and measured by the PCS [36], proved to be an important predictive component of the decision to abandon the test.

Thus far, to our knowledge, there are no studies that have carried out further exploration of the predictors of abandonment of a painful test. A substantial body of research shows that people with low self-efficacy do not tolerate pain well, report greater pain intensity, and experience more interference because of pain [37,38]. In agreement with the previous literature [39], a significant relationship between impulsivity and the level of pain perception emerged in this study. Participants with a higher mean impulsivity orientation reported a higher level of pain perception in the cold box immersion tests.

The analysis of the effect of the manipulation of the attentional focus, introduced as a stimulus in the re-test, did not influence the behavior of abandonment. With respect to the levels of pain perception, a difference was identified within the group with instruction to externalize the focus: the average value of the final perception of pain by those who completed both tests (and in the second trial they received the delivery of externalizing their attention during the immersion of their hand in the cold box) recorded a significant drop in the re-test. This result was consistent with studies from the sports performance sector, in which the effect of the externalizing of attention to fatigue resistance in improving endurance performance, speed, and strength was variously emphasized [40,41,42,43,44,45].

A further contribution of this work is the construction of a general explanatory model of the abandonment behavior of a painful test. In the path analysis model, attentional impulsiveness, task self-efficacy, disposition to catastrophizing, disposition to rumination, and learned helplessness were included as predictor variables. The model, as shown in the results, showed overall good fit measurements, presenting a path of influences that cognitive components exert on behavior, predisposing (or not) to the decision to abandon or give up pursuing a task that involves the endurance of physical pain. Naturally, the experimental procedure used (CPT) implies a reasonable limit to the generalization of the results for non-experimental but natural contexts of acute, clinical and/or chronic pain, to which many scholars have already addressed their attention. However, the model emphasizes the role of cognitive processing (in terms of expectation and forecast scenarios) in the perception and tolerance of physical pain. Researchers have proposed that the magnification and rumination domain stems from a dysfunctional focus and evaluation in the primary appraisal stage, while helplessness is a maladaptive and negative secondary appraisal [46]. The careful consideration of this interweaving between dispositional inclinations and situational processing can be the adequate basis of interventions aimed at supporting the patient in functionally dealing with both acute and chronic pain. Interventions for pain catastrophizing, such as, for example, mindfulness-based interventions, have been found to be feasible, acceptable and efficient non-pharmaceutical treatments for chronic pain management [47].

The path model that was tested in this study revealed that there are two further components on which it is first necessary to work: the self-efficacy of the subject and the weight of previous negative experiences (learned impotence). The first will have a modulating effect on the weight of the second component. Undermining the passive patterns introjected by the participants will also be crucial in blocking the obsessive reactivation of negative thoughts, thus limiting the catastrophic amplification of concerns. The assignment of distracting tasks (corresponding to an externalization of attention) could produce a limiting effect of the motor agitation component in those participants, for whom it has been shown that it reinforces both the catastrophic orientation and the painful perception.

## 5. Strengths and Innovation

No studies have previously been conducted to understand the psychological factors that may predict the abandonment of experimental tests with painful content by the participant. A strong point of this study is the use of the CPT as a reliable method of pain induction with minimal side effects. The main contribution of this work is the construction of a general explanatory model of the abandonment behavior of a test that measures pain tolerance, such as the CPT.

## 6. Limitations

A limitation of this study is that its results cannot be generalized to the non-experimental but natural contexts of acute, clinical and/or chronic pain.

Moreover, the experiment relies on the self-reported declarations of the participants and no objective methods were used to measure the focus on the task.

A further limitation is the age range considered, corresponding to the university population from which the sample was extracted. There was therefore no comparison with older participants, for whom a greater experience with painful episodes could have shown more weight in the final model.

Since only a small number of individuals who abandoned the test were included in the path analysis, this may have impacted the results, constituting an additional limitation of the study.

## 7. Future Studies

In order to strengthen the proposed model and to better assess the abandonment rate, further studies could evaluate the use of a longer time frame of cold immersion, considering that the limit of 3–5 min is normally applied to healthy adults too, while in the present study the limit of 90 s was chosen only as a criterion of maximum caution to avoid any possible risk of tissue damage.

Studies in the future could evaluate the effectiveness of targeted interventions of cognitive and emotional support on the dimensions included in the model described in this study and aim at reinforcing the resistance and persistence in the conduct of tasks that involve the management of physical and mental pain. A further extension of the study could investigate whether the person’s temporal focus could also show associations with the modulation of pain perception [48,49].

A further aspect to be investigated with additional studies could be gender differences associated with the dispositional variables included in the model, in order to understand whether the experimental abandonment behavior of males and females can highlight significant specificities.

## 8. Conclusions

Disposition to catastrophizing, including its components of rumination, amplification, and learned helplessness, proved to be an important predictive component of the decision to abandon a painful task such as the CPT. With respect to the levels of pain perception, the manipulation of the focus did show a difference within the subjects with the externalization of their attention: the average value of the final perception of pain recorded a significant drop in the re-test, while there were no associations with the abandonment behavior of the trials. The general path model of abandonment behavior that was built demonstrated overall good fit measurements. A careful assessment of individuals’ dispositional inclinations and situational processing is important in order to develop specific interventions aimed at supporting people in functionally dealing with both acute and chronic pain.

## Figures and Tables

**Table 1:** Individual differences in experimental abandonments.

Measures	t	*p*	*M_1_*	DS	*M_2_*	DS	95% CI	*d*
*FT Abandonment*								
Impulsiveness	−2.116	0.036	1.75	0.28	1.87	0.33	[−0.245; −0.008]	0.39
Task Self-Efficacy	−2.497	0.013	5.12	0.97	4.61	1.09	[−0.900; −0.104]	0.49
Catastrophizing	−3.16	0.002	2.4	0.48	2.71	0.49	[−0.553; −0.115]	0.64
*ST Abandonment*								
Task Self-Efficacy	−2.819	0.011	5.15	0.9	4.44	1.09	[−1.20; −0.208]	0.71
Catastrophizing	−2.58	0.004	2.4	0.48	2.75	0.59	[−0.619; −0.080]	0.65
*BT Abandonment*								
Task Self-Efficacy	2.182	0.03	4.99	1.26	4.45	1.35	[0.050; 1.02]	0.41
Catastrophizing	−3.13	0.002	2.4	0.87	2.69	0.52	[−0.475; −0.105]	0.4

Note: FT = First Trial; ST = Second Trial; BT = Both Trials; *M_1_* = Mean associated with abandonment; *M_2_* = Mean associated with non-abandonment; SD = Standard Deviation; CI = Confidence Interval; *d* = Cohen’s d. Significant at the *p* < 0.05 level.

**Table 2:** Trait predictors of experimental abandonment.

Measures	F	df	*p*	*ß*	*R^2^*
*FT Abandonment*					
Impulsiveness	1.523	1245	0.22	0.111	0.01
Task Self-Efficacy	0.661	1245	0.418	−0.074	0
Catastrophizing	9.987	1245	0.002	0.276	0.08
*ST Abandonment*					
Impulsiveness	1.941	1245	0.166	0.126	0.02
Task Self-Efficacy	2.055	1245	0.154	−0.129	0
Catastrophizing	8.835	1245	0.004	0.261	0.07
*BT Abandonment*					
Impulsiveness	2.119	1245	0.148	0.131	0.02
Task Self-Efficacy	1.532	1245	0.218	−0.112	0.01
Catastrophizing	11.754	1245	0.001	0.298	0.09

Legend: FT = First Trial; ST = Second Trial; BT = Both Trials; df = degrees of freedom; *ß* = Standardized coefficient Beta. Significant at the *p* < 0.05 level.

**Table 3:** Maximum likelihood estimates and standardized weight estimates.

Label		Label	MLE	S.E.	C.R.	*p*	SWE
Task self-efficacy	→	Learned Helplessness	−0.184	0.039	−4.73	***	−0.394
Learned Helplessness	→	Rumination	0.758	0.094	8.024	***	0.588
Rumination	→	Catastrophizing	0.407	0.036	11.222	***	0.545
Attentional Impulsivity	→	Catastrophizing	0.19	0.059	3.237	0.001	0.127
Learned Helplessness	→	Catastrophizing	0.439	0.047	9.38	***	0.455
Catastrophizing	→	Trials Abandonment	0.47	0.138	3.418	***	0.296

*Note*: MLE: Maximum Likelihood Estimates; SWE: Standardized Regression Weight Estimates. *** *p* < 0.001.

## Data Availability

The datasets during and/or analyzed during the current study are available from the corresponding author on reasonable request.
